# Patient and Family Representation in Randomized Clinical Trials Published in 3 Medical and Surgical Journals: A Systematic Review

**DOI:** 10.1001/jamanetworkopen.2022.30858

**Published:** 2022-09-09

**Authors:** Nissim Benizri, Sophie Hallot, Karen Burns, Michael Goldfarb

**Affiliations:** 1 Division of Internal Medicine, Jewish General Hospital, McGill University, Montreal, Quebec, Canada; 2 McGill Faculty of Medicine and Health Sciences, McGill University, Montreal, Quebec, Canada; 3 Division of Critical Care Medicine, St Michael’s Hospital, University of Toronto, Toronto, Ontario, Canada; 4 Division of Cardiology, Jewish General Hospital, McGill University, Montreal, Quebec, Canada

## Abstract

**Importance:**

Patient and family engagement in research may improve the design, conduct, and dissemination of clinical research, but little is known about whether these stakeholder groups are involved in the design and conduct of randomized clinical trials.

**Objective:**

To characterize the involvement and role of patient and family representatives in the design and conduct of randomized clinical trials by reviewing randomized clinical trials from 3 peer-reviewed medical and surgical journals with high impact factors.

**Evidence Review:**

In this systematic review, the first 50 consecutive randomized clinical trials published on or after January 1, 2021, until September 30, 2021, from each of 3 medical and surgical journals with high impact factors were reviewed for patient or family involvement in trial design and/or conduct. The manuscript, supplemental data, and trial registry records were searched for trial design and governance structures. Two independent, blinded reviewers screened citations and extracted data. This study followed the Preferred Reporting Items for Systematic Reviews and Meta-analyses (PRISMA) guidelines.

**Findings:**

Only 7 of 150 randomized clinical trials (5%) reported patient or family representation in their study design or conduct. Most studies with patient or family representation (n = 5) were from a single journal. Stakeholder involvement was mainly in the execution phase (n = 7), although in 2 studies stakeholders were also involved in the translation phase.

**Conclusions and Relevance:**

The findings of this systematic review suggest that patient or family involvement in the design and conduct of randomized clinical trials in the publications with high impact factors is lacking. We found that when patient or family groups are involved in research, the focus was mainly on the execution phase of research design. There is a need to increase stakeholder involvement in the research design, conduct, and translation of randomized clinical trials.

## Key Points

**Question:**

Are patient or family representative groups involved in the design or conduct of randomized clinical trials published in 3 medical and surgical journals with high impact factors?

**Findings:**

In this systematic review of consecutive randomized clinical trials from 3 journals, 7 of 150 trials (5%) included patient partners or community representative groups. Stakeholder involvement was mainly in the execution phase.

**Meaning:**

These findings suggest that patient or family group involvement in the design and conduct of randomized clinical trials in publications with the highest impact factors is lacking.

## Teaser

This systematic review assesses the involvement and role of patient and family representatives in the design and conduct of randomized clinical trials published in peer-reviewed medical and surgical journals with high impact factors.

## Introduction

Patient and family engagement in health care research is a means to achieve patient-centered health care delivery.^1,2^ There is growing support for patient and family participation in health care research by patient and family advocacy groups, professional societies, and regulatory agencies.^3,4,5^

Opportunities exist for patient and family groups to contribute to health care research throughout the research continuum. Patients or family representatives can collaborate with research personnel and organizations to establish research priorities, refine research questions, assist with study design and protocols, tailor interventions, revise the information provided to study participants, help select study outcomes, and participate in knowledge translation efforts, such as implementation of study findings and practice in community settings.^6,7^ Ultimately, the purpose of patient and family representatives in research is to ensure that research is relevant to patients’ needs.^8^

There is a particular need to include the patient or family perspective in randomized clinical trials. Randomized clinical trials provide some of the strongest evidence of cause and effect in contemporary scientific inquiry and often inform evidence-based guidelines and clinical practice. However, to our knowledge, little is known about whether these stakeholder groups are involved in the design and conduct of randomized clinical trials. In addition, the role of patient and family representatives within randomized clinical trial design committees has not yet been defined. Thus, our objective was to review randomized clinical trials from peer-reviewed medical and surgical journals with high impact factors to characterize the involvement and role of patient and family representatives in clinical trial design.

## Methods

In this systematic review, we reviewed the first 50 consecutive randomized clinical trials published on or after January 1 through September 30, 2021, for each of the following 3 medical and surgical journals with high impact factors: *New England Journal of Medicine* (*NEJM*), *Journal of the American Medical Association* (*JAMA*), and *Annals of Surgery*. Because there were only 32 randomized clinical studies on or after January 1, 2021, in the journal *Annals of Surgery*, we completed the search with 18 consecutive articles published prior to December 31, 2020; the earliest publication date of these 18 articles was July 2020. This study followed the Preferred Reporting Items for Systematic Reviews and Meta-analyses (PRISMA) reporting guideline.

For each study, we searched the main article and supplemental content for information on trial design or management committees. We also reviewed each study to see if it was registered on a clinical trial reporting registry. We searched through the clinical trial registry records to see if we could identify patient or family participation in trial management or design committees that were not reported in the published article or supplemental information. To identify patient, family, or community representative involvement, we searched the table of contents of the trial protocol for trial design or steering or management committees and for keywords such as “patient,” “community,” “investigators,” “representatives,” “advocate,” “family,” and “steering.”

We attempted to contact the corresponding author by email if (1) there was no study design team listed to find out whether patient or family representatives were involved in the study design and role, (2) if a study design team was listed but it was not specified whether there were patient or family representatives involved, or (3) when there was a patient or family member listed in study design but the patient and/or family member role was not clearly defined. A follow-up email was sent to the corresponding author if there was no response to the initial query.

We extracted the following information for each study: author, year, steering group listed, patient and/or family involvement, and role of patient, family, or community group. Studies were categorized into subject areas. Study roles were characterized within the 3 phases of the research process: preparatory (agenda setting and research topic prioritization), execution (study design, recruitment, data collection, and data analysis), and translation (dissemination and implementation).^9^ Two independent, blinded reviewers (N.B. and S.H.) screened studies and extracted data. Disagreements were resolved by a third independent reviewer (M.G.).

Patient, family, or community participation is referred to collectively as patient representation. Summary statistical tests were performed with Microsoft Excel, version 2206 (Microsoft Corp).

## Results

There were 150 studies included in the analysis. Nearly all studies (144 of 150 [96%]) were registered on clinical trial registries. Three studies listed patient representation in their clinical trials registration, but this representation was not included in the published article.^10,11,12^ The most common study categories were general surgery (31 [21%]), COVID-19 (26 [17%]), and medical oncology (15 [10%]) (eTable in the Supplement). Most trials (86 [57%]) had a steering group listed.

There were 7 of 150 randomized clinical trials (5%) that reported patient or family representation in their study design or conduct: 5 from *JAMA* (10%), 1 from *NEJM* (2%), and 1 from *Annals of Surgery* (2%) (Table). All 7 randomized clinical trials with patient involvement included participation in the execution phase, and 2 studies included participation in the translation phase. The study by Befort and colleagues^13^ included a patient advisory board, which was involved with trial design, clinician education, and assisting with the study recruitment process. In their study, DeVore and colleagues^14^ collaborated with a patient engagement committee to participate in recruitment and to ensure that the selected outcomes were patient centered. The study by Drekonja and colleagues^15^ included community representatives to assist with information for study participants and outcome selection. The study by Syversen and colleagues^16^ involved several community organizations in study design, conduct, and dissemination of results. The studies by Ghogawala et al^10^ and Andrade et al^11^ involved patient partners in study design. The study by Pusic and colleagues^12^ included patient partners from a patient advocacy group in study design and conduct.

**Table.** Randomized Clinical Trials With Patient and/or Family Representatives in Their Study Design

Source	Journal	Category	Patient and/or family involvement	Roles
Befort et al,^13^ 2021	*JAMA*	Public health or preventive medicine	REPOWER Rural Patient Advisory Board	Execution Informed trial design Reviewed recruitment, retention, and intervention materials Assisted with clinician trainings Provided a clear patient voice on the way obesity is treated in primary care
DeVore et al,^14^ 2021	*JAMA*	Cardiovascular	Patient Engagement Committee	Execution End point selection: quality-of-life questionnaires; removal of incomplete, redundant, or outcomes of minimal value to people with heart failure Participant recruitment and retention
Drekonja et al,^15^ 2021	*JAMA*	Infection	Community member representatives	Execution Involved in the protocol, consent form, and any subject-facing material (opt-out letters) Involved in creating participant-friendly information End point selection
Syversen et al,^16^ 2021	*JAMA*	Rheumatology	Norwegian Rheumatism Association; Norwegian IBD Patient Organization; Psoriasis and Eczema Association of Norway	Execution Study design: is frequency of follow-up acceptable? End point selection: is outcome important to patients? Study conduct: information and motivation of patients to participate Translation Dissemination of results in webinars
Ghogawala et al,^10^ 2021	*JAMA*	Neurology	Patient partners	Execution Study design
Andrade et al,^11^ 2021	*NEJM*	Cardiovascular	Patient partners	Execution Study design: protocol development Translation Knowledge dissemination
Pusic et al,^12^ 2021	*Annals of Surgery*	General surgery	Patient partners from the patient advocacy group Support Connection	Execution Study design: study materials; constructive feedback Study conduct: recruitment efforts

Abbreviations: IBD, inflammatory bowel disease; *JAMA*, *Journal of the American Medical Association*; *NEJM*, *New England Journal of Medicine*; REPOWER, Rural Engagement in Primary Care for Optimizing Weight Reduction.

## Discussion

Our objective was to evaluate whether patient representatives were involved in randomized clinical trials in medical and surgical journals with high impact factors. We found that only 7 of 150 studies had patient representation, and most of these studies were published in a single journal. Roles for patient representatives varied considerably. Most patient involvement was in the execution phase. This study, to our knowledge, is the first to explore the involvement of patient representatives in the design of randomized clinical trials in the published literature.

Patient and family representation in health research has its origins in community-based participation research. In 2001, the Agency for Healthcare Research and Quality highlighted the need to promote community-based participation research.^1^ Stakeholder engagement in research as a guiding principle was strengthened in 2010 with the founding of the Patient-Centered Outcomes Research Institute (PCORI).^17^ Subsequently, professional societies have supported a role for patient and family participation in the research process.^4^

Stakeholder inclusion in health research is based on the idea that incorporating their perspectives throughout the research process is essential to maintaining the patient’s perspective and to increasing the validity and meaningfulness of the results.^18^ Stakeholder engagement helps to bridge the gap between research, policy, and patient-centered care. Including stakeholders in the research process usually requires an investment of additional resources (ie, time, energy, and money).

Patient and family engagement in research may increase the patient centeredness of study design. In particular, stakeholder engagement may lead to selection of outcome measures that reflect patients’ priorities.^19^ Patients consistently cite their preference for patient-centered outcomes rather than standard clinical or surrogate measures as study end points.^20,21^ One study reported that more than three-fourths of people with diabetes preferred to include patient-centered outcomes that affected their quality of life, such as kidney failure or blindness, rather than surrogate outcomes, such as glycemic control.^22^

Patient involvement has also been shown to increase participant enrollment and retention rates, which could improve study feasibility and the usefulness of results.^9,23^ Patients may have a particular usefulness at improving the readability of informed consent forms and other participant materials.^24^ Patient involvement may also assist with efforts for translational research.^25,26^

Patient representatives can be involved throughout the research process. A systematic review of 142 studies reporting patient involvement in health care research found that patient representatives are most frequently engaged in the execution phase, mainly in study design and protocol development, and less frequently involved in preparatory and translation efforts.^9^ Similarly, all 7 identified randomized clinical trials in our study had patient involvement in the execution phase, while only 2 had patient involvement in the translation phase.

Barriers exist for patient involvement in research. The traditional scientific culture is that research is performed for patients rather than with patients.^27^ Having stakeholders involved in research is a relatively recent concept. Although patient and family involvement in health care delivery is increasingly accepted by professional societies, patient participation in research design and conduct is still not as widely supported.^28^ When patients participate in study design, differing expectations about their roles may exist between patients and researchers.^12^ Patient involvement may require extra time and funding, which can be a barrier to research participation.^9^ There may be ethical limitations to remunerating patients involved in research design and conduct, which may further inhibit some people from participating.^29^ Some areas of research, such as basic sciences, are less focused on patients’ needs, and therefore the patients’ role in this research may be much less significant. In addition, patients may be included in the research team in a tokenistic manner and may not be expected to contribute in a meaningful way.^22^ Some funding agencies, such as PCORI through the US Congress and the Strategy for Patient-Oriented Research of the Canadian Institutes of Health Research, mandate that proposed studies demonstrate meaningful engagement in study design, implementation, and/or knowledge translation to be eligible for funding.^17,30^

Although a broad literature on stakeholder representation exists for observational study design, stakeholders appear to be less commonly involved in randomized clinical trials. Perhaps randomized clinical trials, which are designed to show efficacy or support causality, may require end point selection that is less subject to discussion. Thus, investigators may not feel the need to include stakeholders in their design. Investigators may also feel that stakeholder engagement slows down the research process. The plethora of recent COVID-19 randomized clinical trials that moved quickly from design to recruitment to publication and that did not report stakeholder involvement in our study may support this notion.

Although most randomized clinical trials in our study included a description of the trial design and management committees, there were some studies that did not. It is unclear whether this lack of reporting is reflective of a lack of stakeholder inclusion in study design and governance or due to deficiencies in research reporting. A reporting guideline under development, CRISP (Checklist for Reporting Research Using Simulated Patient Methodology), calls explicitly for reporting patient, family, and community member involvement in health care research.^31^

### Limitations

There were limitations to our study. First, only 3 journals were included, limiting generalizability. It is possible that the randomized clinical trials published in these journals may not be the most likely to include patient and family representation and that randomized clinical trials in other journals may have a higher percentage of patient and family representation. However, the selected journals are among the top-ranked journals in the medical and surgical fields and often inform clinical practice guidelines. Second, the study timeframe included the COVID-19 pandemic, and COVID-19–related trial protocols may have been developed in an expedited manner. Many of these randomized clinical trials were likely published in an expedited manner to provide more rapid insight into effective therapeutic strategies and may not have had the opportunity to incorporate patient involvement in the design or conduct. Third, many trials did not include information about their steering or oversight committees. We were able to contact authors from 29 of 67 studies that did not include this information, and only 1 of 29 contacted authors (3%) reported patient involvement in the trial design. Thus, it is unlikely that there are many more studies with patient involvement in study design.

## Conclusions

The findings of this systematic review suggest that patient or family group involvement in the design and conduct of randomized clinical trials in publications with the highest impact factors is lacking. This study found that when patient or family groups are involved in research, the focus was mainly on the execution phase of research design. There is a need to increase stakeholder involvement in research design, conduct, and translation.

## Notes

Supplement. **eTable.** Randomized Clinical Trial Subject Areas and Frequency
