# Supplementary material for: Patient and Family Representation in Randomized Clinical Trials Published in 3 Medical and Surgical Journals: A Systematic Review
Source: JAMA Netw Open. 2022 Sep 9;5(9):e2230858. doi: 10.1001/jamanetworkopen.2022.30858 (PMC9463605; doi:10.1001/jamanetworkopen.2022.30858)
Supplement: Supplement. — eTable. Randomized Clinical Trial Subject Areas and Frequency [file jamanetwopen-e2230858-s001.pdf]

Supplementary Online Content

Benizri N, Hallot S, Burns K, Goldfarb M. Patient and family representation in randomized clinical trials published in 3 medical and surgical journals: a systematic review. *JAMA Netw Open.* 2022;5(9):e2230858. doi:10.1001/jamanetworkopen.2022.30858

eTable. Randomized Clinical Trial Subject Areas and Frequency

This supplementary material has been provided by the authors to give readers additional information about their work.

© 2022 Benizri N et al. *JAMA Network Open.*

eTable. Randomized Clinical Trial Subject Areas and Frequency

**Subject areas**	**Studies (N=; %)**
General surgery	31 (21%)
COVID-19	26 (17%)
Medical oncology	15 (10%)
Cardiovascular	13 (9%)
Neurological	10 (7%)
Infection	9 (6%)
Rheumatological	6 (4%)
Psychiatry/ Psychology	6 (4%)
Hematology	5 (3%)
Respiratory disease	5 (3%)
Nephrology	4 (3%)
Gastroenterology	4 (3%)
Obesity	4 (3%)
Endocrinology	3 (2%)
Dermatology	3 (2%)
Obstetrics/Gynecology	2 (1%)
Vascular Surgery	2 (1%)
Public health and Preventive Medicine	2 (1%)

© 2022 Benizri N et al. *JAMA Network Open.*
